# Feasibility and Biomechanical Effects of Dynamic Neuromuscular Stabilization Training During Stair Negotiation in Middle-Aged Women with Knee Osteoarthritis: A Randomized Controlled Pilot Study

**DOI:** 10.3390/jfmk11030255

**Published:** 2026-06-27

**Authors:** Hyun Ju Kim, Shu Ho Kang, Young Joo Cha, Il Bong Park

**Affiliations:** 1 Department of Sports Medicine, Busan University of Foreign Studies, Busan 46234, Republic of Korea; 20246204@bufs.ac.kr (H.J.K.); 20236204@bufs.ac.kr (S.H.K.); 2 Department of Physical Therapy, Cheju Halla University, Jeju 63092, Republic of Korea; chazoo0849@chu.ac.kr

**Keywords:** knee osteoarthritis, exercise therapy, postural control, center of pressure, lateral stability, lower-limb kinematics

## Abstract

**Background**: Knee osteoarthritis (KOA) alters the performance of daily activities, such as stair negotiation, by compromising lateral stability and neuromuscular control. This pilot study aimed to evaluate the feasibility of a 10-week Dynamic Neuromuscular Stabilization (DNS) program and to explore its preliminary biomechanical effects during stair ascent and descent in middle-aged women with KOA. **Methods**: Twenty-six participants were randomly assigned to a DNS group (*n* = 13) or a control group (*n* = 13). The DNS group completed a 10-week intervention (twice weekly). Feasibility was assessed via recruitment, retention, and adherence. Primary outcomes were mediolateral (ML) center of pressure (COP) parameters, while secondary outcomes included anteroposterior (AP) COP parameters and lower limb range of motion (ROM). Effect sizes (η^2^_p_) were estimated using 3D motion analysis and force plates. **Results:** The intervention showed high potential feasibility, with 100% recruitment and retention rates and 98.5% compliance. No adverse events occurred. Large effect sizes were observed for reduced ML COP velocity (ascent: η^2^_p_ = 0.79; descent: η^2^_p_ = 0.62) and RMS (descent: η^2^_p_ =0.16). Secondary outcomes, including AP COP parameters and joint ROM (increased sagittal flexion and decreased coronal instability), also demonstrated large effect sizes. **Conclusions**: This pilot study suggests that progressive DNS training appears to be feasible and safe for patients with KOA. The preliminary effect sizes observed in COP control and lower kinetic chain mechanics may serve as useful foundational data for designing future adequately powered clinical trials to further examine the efficacy and underlying biomechanical mechanisms of DNS training.

## 1. Introduction

Knee osteoarthritis (KOA) is a prevalent musculoskeletal disorder affecting approximately 23% of the global population aged 40 and older. It significantly threatens the independence of daily living, particularly in middle-aged women, who exhibit an overwhelmingly higher prevalence than men [1,2,3]. Consequently, these female patients experience significant declines in gait performance and elevated biomechanical burden not only during level walking but also during stair negotiation, an essential activity of daily living [4,5,6].

Stair ascent and descent generate joint loads reaching 3–4 times body weight [7,8,9] and have been reported to require a wider lower-limb range of motion (ROM) and a high degree of dynamic postural control [10,11,12]. Specifically, because stair descent necessitates eccentric contraction to control weight-bearing, patients with KOA adopt altered gait strategies due to pain and muscle weakness, which ultimately leads to a substantial decrease in dynamic stability [13,14].

In evaluating the dynamic stability of stair negotiation, the mediolateral (ML) velocity of the center of pressure (COP) is considered a primary indicator that sensitively reflects functional deficits in patients with KOA [15,16]. While anteroposterior (AP) movement during stair walking is significantly constrained by the physical structure of the step tread [17], ML stability depends largely on the individual’s neuromuscular control [18].

Furthermore, because KOA patients experience biomechanical structural deformation (varus thrust) due to cartilage damage in the medial compartment of the knee, they tend to exhibit reduced lateral control against outward pushing forces during weight-bearing [19,20,21]. Additionally, as a compensatory mechanism to avoid pain, they exhibit a pattern of using their lower extremities stiffly, which induces an abnormal decrease in the lower-limb joint ROM essential for shock absorption and propulsion [9,12,22,23]. Therefore, to improve the stair-negotiation ability of KOA patients, an intervention that restores deep muscle recruitment and neuromuscular control, rather than mere muscle strengthening, is critically required.

Previous exercise-intervention studies have attempted to improve functional performance and postural control in patients with KOA through task-specific training, balance-based exercise, and external loading strategies. For example, stair-climbing training, Tai Chi, balance/postural control training, and unstable load training have been reported to improve physical activity, spatiotemporal stair-gait parameters, dual-task performance, lower-extremity kinematics, COP control, and pain in patients with KOA [24,25,26]. Although these interventions have demonstrated beneficial effects, an intervention that more directly targets deep neuromuscular control and trunk–lower limb coordination may be required to further improve dynamic control during stair negotiation.

DNS training is an exercise technique based on developmental kinesiology that reactivates the motor control programs of the central nervous system and strengthens the deep stabilizing system [27,28,29]. Recent studies have reported that DNS training can enhance core stability and improve lower extremity alignment, thereby enhancing both static and dynamic balance [27,28,29,30]. This improvement in core-based anticipatory postural control ultimately provides a solid base of support for active limb movements, which is expected to have a positive impact even when performing more complex dynamic tasks.

However, despite these positive findings from previous studies, research analyzing the effects of DNS training on the dynamic biomechanical changes during ‘stair negotiation’, a task with high functional demands for KOA patients, is still lacking. In particular, studies quantitatively evaluating the ML COP velocity control ability during stair walking—which is closely related to lateral stability—and the changes in lower-limb joint ROM required for task execution have rarely been conducted.

Therefore, this pilot study aimed to evaluate the feasibility of a 10-week DNS training program and to explore its biomechanical effects during stair ascent and descent in middle-aged women with knee osteoarthritis. ML COP velocity and RMS were selected as primary outcomes of lateral dynamic stability, and AP COP parameters and lower-limb ROM were included as secondary outcomes.

## 2. Materials and Methods

### 2.1. Participants

This pilot study targeted middle-aged women aged 50 to 64 years who were diagnosed with KOA. Only women were included in this pilot study to reduce sex-related heterogeneity in biomechanical and neuromuscular outcomes and because KOA is more prevalent and often more symptomatic in middle-aged and older women [4,5,6]. Following the sample size recommendations for pilot studies [31,32], and accommodating a potential dropout rate of 10–15%, a total of 26 participants (*n* = 13 per group) were recruited. This ensures sufficient data to estimate effect sizes and conduct exploratory analyses for future definitive trials. Participants were recruited through announcements posted on bulletin boards at the Lifelong Education Center and the Sports Rehabilitation Laboratory of Busan University of Foreign Studies.

The eligibility criteria were divided into inclusion and exclusion criteria, as described below.

The inclusion criteria were as follows:

- Individuals diagnosed with KOA (Kellgren–Lawrence grade II–III) by an orthopedic surgeon based on radiographic Kellgren–Lawrence grading and recommended for exercise therapy, verified through medical documentation for participant safety.
- Individuals with no clinical history of musculoskeletal disorders other than KOA.
- Individuals capable of independent stair negotiation without gait aids.
- Individuals who voluntarily provided written informed consent after receiving a full explanation of the study procedures.

The exclusion criteria were as follows:

- Intra-articular knee injections within the previous 3 months.
- A history of knee joint surgery.
- Severe cardiovascular, neurological, or psychiatric disorders.
- Acute inflammation or intractable pain preventing exercise performance.
- Other significant medical conditions, such as chronic respiratory disease or major surgery within the previous 6 months.

The 26 eligible participants were randomly allocated in a 1:1 ratio to either the experimental group (DNS training, *n* = 13) or the control group (activities of daily living, *n* = 13) using simple randomization. The random allocation sequence was generated by an independent researcher who was not involved in participant recruitment, assessment, or intervention. Group assignments were placed in sequentially numbered, opaque, sealed envelopes to ensure allocation concealment. After baseline measurements were completed, each participant selected or was assigned the next envelope in sequence, and the group allocation was revealed. A single-blind protocol was implemented, in which the outcome assessors remained unaware of group assignments during all measurements. Following ethical guidelines, a wait-list control design was used; the control group was offered an 8-week DNS exercise program (twice weekly) immediately after the 10-week study period. This study was approved by the Public Institutional Review Board designated by the Ministry of Health and Welfare (Approval No.: P01-202511-01-012, approved on 10 November 2025) and was registered with the Clinical Research Information Service (CRIS) (Registration date: 13 November 2025; Registration No.: KCT0011158). All participants received a thorough explanation of the purpose and procedures of the study and voluntarily provided written informed consent prior to participation.

**Figure 1:**
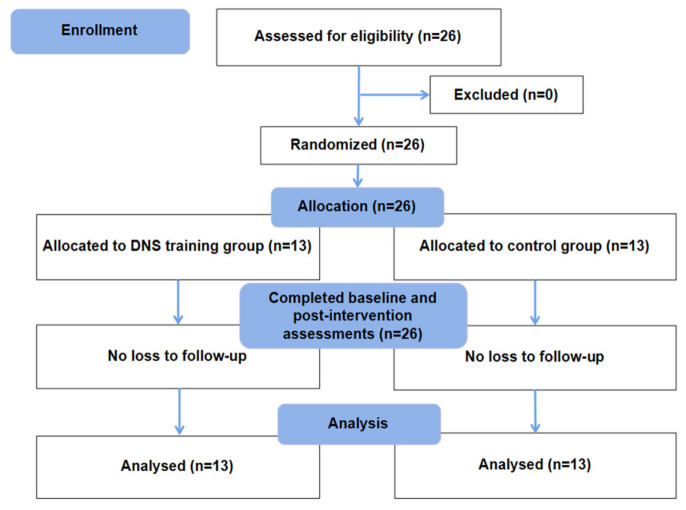
Flow diagram of the study participants.

Baseline demographic characteristics and the CONSORT flow diagram are presented in Table 1 and Figure 1, respectively.

### 2.2. Stair Ambulation Analysis

In this study, the ML velocity and RMS of the COP, representing lateral dynamic stability, were established as the primary outcomes. Additionally, the sagittal and coronal ROM of the lower-limb joints and AP COP parameters were analyzed as secondary outcomes, while joint moments were evaluated as supplementary kinetic outcomes.

**Figure 2:**
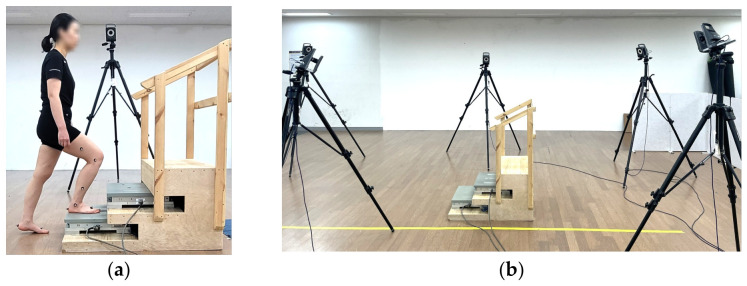
Biomechanical measurement environment and experimental equipment. (**a**) Marker placement and the transition movement of the participant. (**b**) Three-step staircase structure integrated with the motion analysis system and force plates.

To evaluate these variables, a custom-built three-step staircase, constructed in accordance with Korean standard building codes (riser height: 17 cm), was utilized. To capture kinetic data, two force plates (AMTI OR6, Watertown, MA, USA) were directly embedded into the first and second steps (Figure 2).

The dimensions of all steps were identical across the staircase, ensuring standardized measurement conditions for all participants. Three-dimensional kinematic data were recorded at 100 Hz using the Vicon Plug-in Gait system (Vicon camera MX-T20, Oxford Metrics, Oxford, UK), with 16 retroreflective markers attached to specific anatomical landmarks of the lower extremities, synchronously with ground reaction force data sampled at 1000 Hz. The kinematic and kinetic data were processed using Vicon Nexus software, version 2.15, with the Plug-in Gait model (Vicon camera MX-T20, Oxford Metrics, Oxford, UK). To calculate the kinetic variables, data from the force plate that the participants first contacted during both stair ascent and descent were used for analysis. Prior to calculating the biomechanical variables, the raw data were processed using a zero-lag, fourth-order Butterworth low-pass filter with a cutoff frequency of 10 Hz [33,34].

Prior to data collection, participants were allowed sufficient practice trials to familiarize themselves with the experimental setup. Participants performed both stair ascent and stair descent tasks barefoot at a self-selected, comfortable pace. For each task, five trials were initially recorded. Subsequently, three successful trials that met the predetermined criteria for consistent and artifact-free execution were selected, and their average was used for the final analysis. To minimize potential measurement and analysis bias, the same marker placement protocol, calibration procedure, filtering method, outcome definitions, and trial selection criteria were applied consistently to all participants. In addition, the outcome assessors remained blinded to group allocation during data collection. Based on these filtered trajectory and force data, the COP was extracted. To account for individual variations, COP velocity was normalized by dividing it by each participant’s gait speed, resulting in a dimensionless ratio. Furthermore, lower extremity joint kinematics and internal joint moments were analyzed using the Plug-in Gait model. Specifically, the ROM was calculated as the difference between the maximum and minimum joint angles in the sagittal and frontal planes. Internal joint moments were also analyzed using their maximum peak values in the same planes; detailed data for joint moments are provided as a Supplementary Material. The stance phase was defined as the continuous period from initial foot strike to foot-off on the embedded force plates.

Due to the bilateral nature of the symptoms, the more symptomatic leg, determined by a higher Visual Analog Scale (VAS) pain score, was designated as the index limb for the stair ambulation analysis [35,36]. As a safety precaution, bilateral handrails were installed on the customized staircase, and an evaluator closely monitored all trials. To prevent any confounding effects on the biomechanical data, participants were instructed to perform the tasks without utilizing the handrails. Ultimately, all participants successfully completed the tasks independently without any safety incidents.

### 2.3. Intervention and Control Conditions

The intervention group completed a 10-week, bi-weekly DNS exercise program (50 min/session). Based on developmental kinesiology, the protocol focused on postural alignment and intra-abdominal pressure (IAP) regulation. Exercises were modified to ensure optimal neuromuscular activation and minimal joint stress for female patients with knee osteoarthritis [27,37].

Training occurred in small groups (n = 6–7) under the direct supervision of an experienced DNS-certified trainer, ensuring high exercise fidelity through continuous feedback. Correct diaphragmatic breathing and proper IAP regulation were verified using both visual observation of the abdominal wall and tactile feedback (e.g., palpation of the lower and posterolateral abdomen) to ensure symmetrical expansion. Optimal joint centration was strictly monitored, and immediate verbal and tactile cues were provided to correct any compensatory strategies, such as rib flaring or loss of neutral spinal alignment.

To safely accommodate female patients with knee osteoarthritis experiencing varying levels of pain and limited range of motion, patient-specific clinical adaptations were applied to minimize joint stress. First, high-density, thick mats were utilized during floor-based exercises to reduce patellofemoral contact pressure. Second, the base of support for the lower extremities was individually widened to enhance stability depending on the participant’s balance and comfort. Third, for participants experiencing discomfort during deep knee flexion or seated postures, yoga blocks were strategically placed under the hips. This modification significantly reduced the knee flexion angle, alleviating patellofemoral joint stress while allowing participants to successfully perform the core DNS tasks. Furthermore, exercises requiring direct knee-weight-bearing (e.g., quadruped postures and subsequent transitional movements) were systematically delayed and introduced progressively in the later phases of the program.

The progression criteria for advancing to more complex or weight-bearing kinematic positions (e.g., from lateral recumbent to upright postures) were strictly defined based solely on movement competence, rather than time. Participants were permitted to progress to the next postural level only when they demonstrated the ability to maintain proper IAP regulation, optimal joint centration, and neutral spinal alignment for the prescribed repetitions or breath cycles without experiencing knee pain or relying on compensatory movements. Intensity was systematically progressed by advancing to more complex kinematic positions (e.g., from lateral recumbent to weight-bearing upright postures), permitted only when participants maintained proper IAP control and joint centration without pain or compensation.

Conversely, participants assigned to the control group did not receive the DNS intervention and were instructed to maintain their habitual lifestyle and daily activities throughout the 10-week study period. To isolate the effects of the intervention, all participants were prohibited from initiating any new structured exercise programs. To ensure safety and ethical compliance, researchers continually monitored for medical consultations, medication use, or significant lifestyle changes. While incidental physical therapy was permitted if prescribed for medical necessity, no participants in either group reported seeking additional therapeutic interventions or experiencing exacerbation of knee pain during the study. Consequently, no confounding variables related to external treatments were introduced into the analysis.

**Figure 3:**
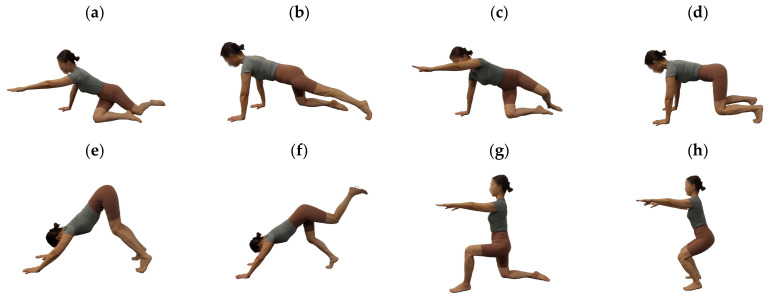
Representative exercises of the 10-week Dynamic DNS program. (**a**) Asymmetrical kneeling transition with contralateral reach; (**b**) Unilateral hip extension with core stabilization in four-point kneeling; (**c**) Contralateral reciprocal crawling pattern; (**d**) Closed-kinetic chain stabilization in levitated four-point kneeling; (**e**) Transition to high plantigrade quadruped position for posterior chain elongation; (**f**) Unilateral lower extremity lift in plantigrade quadruped position; (**g**) Transition from asymmetrical sitting to unilateral weight-bearing lunge; (**h**) Closed-kinetic chain squatting from plantigrade quadruped posture.

The exercise components are illustrated in Figure 3, and the specific set configurations are summarized in Table 2.

### 2.4. Statistical Analysis

All statistical analyses were performed based on the intention-to-treat (ITT) principle. Given the 100% retention rate and the absence of protocol violations, the ITT population was identical to the per-protocol (PP) population.

All data collected in this study were analyzed using IBM SPSS Statistics for Windows, version 25.0 (IBM Corp., Armonk, NY, USA). Descriptive statistics were presented as means and standard deviations for all variables. To verify the homogeneity of demographic characteristics and baseline variables between the groups, an independent *t*-test was performed. Prior to the main analyses, the Shapiro–Wilk test was utilized to assess the normality of the data distribution.

To evaluate the intervention effects, an Analysis of Covariance (ANCOVA) was initially conducted, with baseline values entered as covariates. Prior to conducting the ANCOVA, the assumption of homogeneity of regression slopes was tested. For variables that satisfied this assumption, a standard ANCOVA was applied to determine the differences between the groups. Conversely, for a subset of joint moment variables in the Supplementary Material that violated this assumption, the intervention effect was analyzed using change scores (post-intervention minus baseline values) via independent *t*-tests. Effect sizes were reported as partial eta squared (η^2^_p_) for ANCOVA and Cohen’s *d* for *t*-tests (small: η^2^_p_ = 0.01, *d* = 0.20; medium: η^2^_p_ =0.06, *d* = 0.50; large: η^2^_p_ =0.14, *d* = 0.80).

The Benjamini–Hochberg False Discovery Rate (FDR) procedure was used to correct for multiple comparisons among the secondary outcomes, and the level of statistical significance was set at *p* < 0.05 for all analyses.

## 3. Results

The results of the ANCOVA comparing between-group differences in the primary and secondary outcomes, with baseline values entered as covariates, are presented in Table 3 and Table 4, Table 5 and Table 6, respectively. Additional data regarding joint moments are provided in the Supplementary Material (Tables S1 and S2).

### 3.1. Feasibility, Retention, and Intervention Adherence

In accordance with the primary objectives of this pilot study, feasibility was evaluated through the recruitment rate, participant retention, adherence to the intervention program, the incidence of adverse events, and assessment completion. All 26 eligible individuals agreed to participate, achieving a 100% recruitment rate. Consequently, all 26 randomized participants successfully completed the 10-week study period, resulting in a 100% retention rate and a 0% dropout rate. Participants in the DNS experimental group demonstrated excellent adherence to the 20-session (twice a week) program. Specifically, 11 out of the 13 participants (84.6%) attended all sessions, with only two individuals missing two sessions each due to personal reasons. As a result, the overall compliance rate was 98.5% (256 out of 260 total expected sessions). Furthermore, no adverse events, such as exercise-related injuries or exacerbation of knee pain, occurred throughout the study period, and all participants successfully completed the biomechanical assessments without any technical issues, missing data or dropouts. Taken together, these findings suggest that the applied DNS program was feasible, acceptable, and appeared safe in this pilot sample.

### 3.2. Primary Outcomes

#### Mediolateral COP Velocity and RMS During Stair Ascent and Descent

During stair ascent, ANCOVA revealed a group effect on ML-velocity (*F* = 84.34, *p* < 0.001, η^2^_p_ = 0.79; MD = −0.05 cm/s, 95% CI: −0.06 to −0.04). For ML-RMS, the group effect was *F* = 3.91, *p* = 0.060, η^2^_p_ = 0.15 (MD = −0.24 cm, 95% CI: −0.49 to 0.01).

During stair descent, ANCOVA revealed a group effect on ML-velocity (*F* = 37.37, *p* < 0.001, η^2^_p_ = 0.62; MD = −0.05 cm/s, 95% CI: −0.07 to −0.04). For ML-RMS, the group effect was *F* = 4.46, *p* = 0.046, η^2^_p_ = 0.16 (MD = −0.20 cm, 95% CI: −0.386 to −0.004).

### 3.3. Secondary Outcomes

#### 3.3.1. Anteroposterior COP Velocity and RMS During Stair Ascent and Descent

During stair ascent, ANCOVA revealed a group effect on AP-velocity (*F* = 1.39, *p* = 0.250, FDR-adjusted *p* = 0.250, η^2^_p_ = 0.06; MD = 0.01 cm/s, 95% CI: −0.01 to 0.03). For AP-RMS, the group effect was *F* = 5.60, *p* = 0.027, FDR-adjusted *p* = 0.054, η^2^_p_ = 0.20 (MD = −0.58 cm, 95% CI: −1.08 to −0.07).

During stair descent, the group effect on AP-velocity was *F* = 2.30, *p* = 0.143, FDR-adjusted *p* = 0.143, η^2^_p_ = 0.09 (MD = −0.04 cm/s, 95% CI: −0.09 to 0.01). For AP-RMS, the group effect was *F* = 4.43, *p* = 0.047, FDR-adjusted *p* = 0.094, η^2^_p_ = 0.16 (MD = −0.59 cm, 95% CI: −1.18 to −0.01).

#### 3.3.2. Sagittal and Coronal Joint Range of Motion During Stair Ascent

For sagittal ROM, the group effects were: hip (*F* = 6.33, *p* = 0.019, FDR-adjusted *p* = 0.029, η^2^_p_ = 0.23; MD = 4.01°, 95% CI: 0.71 to 7.30), knee (*F* = 24.60, *p* < 0.001, FDR-adjusted *p* < 0.001, η^2^_p_ = 0.52; MD = 9.70°, 95% CI: 5.65 to 13.74), and ankle (*F* = 11.26, *p* = 0.003, FDR-adjusted *p* = 0.006, η^2^_p_ = 0.33; MD = 2.92°, 95% CI: 1.12 to 4.72).

For coronal ROM, the group effects were: hip (*F* = 3.96, *p* = 0.059, FDR-adjusted *p* = 0.071, η^2^_p_ = 0.15; MD = −1.27°, 95% CI: −2.59 to 0.05), knee (*F* = 14.47, *p* = 0.001, FDR-adjusted *p* = 0.003, η^2^_p_ = 0.39; MD = −3.85°, 95% CI: −5.94 to −1.75), and ankle (*F* = 0.29, *p* = 0.595, FDR-adjusted *p* = 0.595, η^2^_p_ = 0.01; MD = −0.37°, 95% CI: −1.76 to 1.04).

#### 3.3.3. Sagittal and Coronal Joint Range of Motion During Stair Descent

For sagittal ROM, the group effects were: hip (*F* = 7.84, *p* = 0.010, FDR-adjusted *p* = 0.020, η^2^_p_ = 0.25; MD = 2.81°, 95% CI: 0.74 to 4.89), knee (*F* = 33.12, *p* < 0.001, FDR-adjusted *p* < 0.001, η^2^_p_ = 0.59; MD = 9.87°, 95% CI: 6.32 to 13.41), and ankle (*F* = 6.43, *p* = 0.018, FDR-adjusted *p* = 0.027, η^2^_p_ = 0.22; MD = 3.95°, 95% CI: 0.73 to 7.18).

For coronal ROM, the group effects were: hip (*F* = 5.91, *p* = 0.023, FDR-adjusted *p* = 0.028, η^2^_p_ = 0.21; MD = −2.31°, 95% CI: −4.28 to −0.35), knee (*F* = 16.56, *p* < 0.001, FDR-adjusted *p* < 0.001, η^2^_p_ = 0.42; MD = −2.27°, 95% CI: −3.43 to −1.12), and ankle (*F* = 4.50, *p* = 0.045, FDR-adjusted *p* = 0.045, η^2^_p_ = 0.16; MD = −0.69°, 95% CI: −1.37 to −0.02).

### 3.4. Supplementary Outcomes

#### Sagittal and Coronal Joint Moments

Detailed statistical data for the internal joint moments of the hip, knee, and ankle in the sagittal and coronal planes are provided in the Supplementary Material (Tables S1 and S2). Following FDR correction, no group effects were found during stair ascent. During stair descent, group effects were observed on the sagittal knee moment (*F* = 7.53, *p* = 0.012, η^2^_p_ = 0.25) and the sagittal ankle moment (*F* = 9.38, *p* = 0.006, η^2^_p_ = 0.29).

## 4. Discussion

The primary objective of this pilot study was to preliminarily explore the effects of a 10-week DNS intervention on COP characteristics and lower extremity biomechanical variables during stair walking in women with knee osteoarthritis. While previous DNS studies have primarily focused on core stability, chronic low back pain, or static balance, the present pilot study is meaningful in that it expands the clinical application of DNS by analyzing lower extremity coordination during a highly demanding dynamic task. In particular, by adopting stair walking as the experimental task, which requires high-load weight-bearing and complex multi-planar control, this study specifically explored how proximally secured stability may be transferred to the lower extremity kinetic chain. Consequently, the positive trends observed in COP and lower extremity kinematics support the possibility that the principle of joint centration in DNS may help optimize force transfer throughout the kinetic chain. Ultimately, these findings preliminarily suggest that DNS training may contribute to improving movement control strategies and dynamic stability during stair walking in patients with knee osteoarthritis.

In this pilot study, ML COP parameters (velocity and RMS) were designated as primary outcomes, whereas AP COP parameters and lower extremity joint ROM were established as secondary outcomes. Given the pilot design, data interpretation focused on the direction of the estimated effects and effect size estimates. The primary outcomes, ML COP velocity and RMS, showed a decreasing tendency after the intervention, and these changes were accompanied by relatively large effect size estimates. In addition, the lower extremity kinematic variables showed a consistent pattern of increased sagittal ROM and reduced coronal ROM. However, given the small sample size of this pilot study, these effect size estimates should be interpreted as preliminary and exploratory.

Stair ascent involves overcoming gravity to propel the body upward, which requires firm support during the single-leg support phase [38]. The large effect sizes for the reductions in ML COP velocity and RMS observed during the ascent task indicate that post-training, the subjects experienced decreased magnitude and speed of lateral sway. According to previous studies, DNS training establishes adequate IAP through the coordination of respiration and deep muscles, securing robust proximal stability of the trunk [30,37]. Based on these biomechanical principles, a plausible explanation is that subjects utilized the trunk as a more stable anchor point to regulate lateral excursion during step-up movements. However, without electromyography (EMG) or direct assessment of co-contraction, we cannot definitively claim that this represents an optimized neuromuscular strategy. The reduced ML sway might indeed reflect improved dynamic control, but it could alternatively reflect a more constrained, protective movement strategy adopted by the subjects to limit joint loading.

Stair descent is a mechanically demanding task that requires controlling the downward acceleration of the body and safely accepting the load at the moment of foot contact [39,40]. During this process, patients with osteoarthritis frequently employ a compensatory strategy of excessive lateral trunk lean to avoid knee pain and instability caused by weight-bearing [13,41]. The substantial reductions in ML COP velocity and RMS observed during descent suggest that participants may have attenuated this compensatory lateral sway. Theoretically, the proximal stability cultivated through DNS training might have facilitated better management of the body’s downward acceleration and dynamic perturbations upon landing. Nevertheless, the clinical meaning of these COP alterations must be interpreted cautiously. Because subjective pain, perceived difficulty, muscle strength, and functional performance outcomes were not directly assessed, it is difficult to confirm whether this reduction in sway indicates true neuromuscular efficiency or a stricter, guarded movement pattern to avoid discomfort. Therefore, while these findings suggest potentially favorable adaptations in postural control, the underlying mechanistic claims should be viewed as plausible hypotheses rather than confirmed pathways.

In the present study, the sagittal ROM of the joints during stair ascent increased with large effect sizes, whereas the coronal ROM of the hip and knee joints decreased. Previous studies have noted that patients with knee osteoarthritis often employ a protective stiffening strategy, restricting sagittal flexion to avoid pain during stair ascent, while compensating by excessively leaning the trunk or increasing coronal plane movements [5,22]. In this context, the increased sagittal ROM observed post-intervention may suggest a promising shift toward the functional flexion–extension mechanics necessary for upward propulsion. However, without direct assessments of subjective pain, muscle strength, or broader functional outcomes, it is difficult to determine whether this change reflects improved neuromuscular control or a meaningful reduction in protective stiffening strategies. The decrease in coronal ROM presents a similar interpretive nuance. While this reduction may indicate improved frontal-plane stability, consistent with the DNS principle of joint centration, it may also reflect a more constrained or protective strategy used to limit lateral excursion. Consequently, these kinematic changes highlight meaningful movement adaptations, but they should be viewed as potentially favorable responses rather than confirmed mechanisms of enhanced neuromuscular efficiency or reduced joint loading.

Conversely, stair descent is a demanding eccentric control task that requires regulating downward acceleration and absorbing impact at the moment of foot contact [39]. Due to the fear associated with weight acceptance, patients with osteoarthritis tend to abnormally increase joint stiffness to counteract the impact [22,42]. In this context, the increase in sagittal ROM observed during descent may suggest that participants used greater lower-limb joint excursion to accommodate body lowering and impact absorption, rather than relying solely on a rigid landing pattern. Concurrently, the reduced coronal ROM may indicate improved frontal-plane control during landing; however, it could also reflect a more constrained or protective descent strategy. From a theoretical perspective, DNS training may have contributed to improved proximal postural control through IAP regulation and joint centration, which could have influenced lower-limb movement patterns during stair descent [27,28,37,43]. The supplementary kinetic findings, including reduced hip and knee moments with an increased ankle moment, may further suggest a possible redistribution of lower-limb joint demands during descent. However, these interpretations remain exploratory. Because this study did not include direct physiological measurements, such as electromyography, muscle strength testing, or assessment of co-contraction, the underlying neuromuscular mechanisms cannot be confirmed. Therefore, these combined kinematic and kinetic changes should be regarded as plausible biomechanical adaptations associated with DNS training, rather than definitive evidence of reduced joint loading or enhanced neuromuscular efficiency.

In summary, progressive DNS training, based on developmental kinesiology stages, systematically establishes IAP through the coordination of respiration and deep muscles. The proximal stability achieved through this process provides a biomechanical foundation that enables the lower kinetic chain to coordinate synergistically in regulating lower-limb joint movements [37,44]. Consequently, this conceptual framework offers a plausible explanation for the kinematic and kinetic shifts observed during stair negotiation: the reduction in COP sway, coupled with increased sagittal-plane ROM and restricted coronal-plane movement. While these findings provide preliminary evidence that DNS interventions can significantly alter movement strategies in patients with knee osteoarthritis, we must refrain from drawing definitive conclusions regarding enhanced neuromuscular efficiency or fully mitigated mechanical vulnerabilities. Future studies incorporating electromyography, muscle strength testing, and subjective clinical outcomes are necessary to determine whether these kinematic adaptations represent true functional optimization or protective movement strategies.

When interpreting the results of this study, the following limitations should be considered. First, regarding the study design, this was a small-scale pilot study restricted to women with knee osteoarthritis, which limits the generalizability of the findings. In addition, the use of a usual-care wait-list control group is an important limitation. Although acceptable for a pilot study, this design did not control for therapist attention, supervised exercise exposure, participant expectations, or social interaction. Therefore, some between-group differences may reflect non-specific effects of supervised participation rather than DNS-specific effects, and the intervention effect size may have been somewhat overestimated. Furthermore, the relatively large partial eta-squared values observed in this study should be interpreted cautiously, because effect size estimates derived from a small pilot sample may be unstable and may overestimate the likely effects in a future fully powered trial. Accordingly, these estimates should be regarded as preliminary and exploratory rather than confirmatory. The adjusted between-group differences and variability estimates obtained in this study, particularly for the primary outcome, will be used to inform sample-size calculation for a future fully powered randomized controlled trial.

Methodological limitations concerning the experimental setup and measurements also exist. Due to the structural constraints of the 3-step experimental staircase, it was difficult to extract data from a continuous steady-state gait phase. Consequently, the present results primarily reflect the mechanical characteristics of the transition phase from level walking to stair negotiation, rather than steady-state stair walking. Moreover, this study did not directly verify via surface electromyography (sEMG) whether the observed biomechanical changes were attributable to alterations in muscle co-contraction or neuromuscular control. Finally, to ensure the accuracy of marker tracking, the experiment was conducted under barefoot conditions. Although this condition improved marker visibility and standardized the measurement environment, it may reduce ecological validity because individuals typically negotiate stairs while wearing footwear in daily life. In addition, the instruction not to use handrails may differ from real-life stair negotiation, particularly in individuals with knee osteoarthritis who may rely on handrails for stability or pain avoidance. Therefore, the present findings should be generalized cautiously to everyday stair-negotiation environments.

Based on these limitations, several directions for future research can be suggested. First, larger-scale randomized controlled trials including both women and men with knee osteoarthritis are needed to confirm the effect size and generalizability of DNS training. In addition, future studies should consider including an active control group, such as conventional strengthening exercise, to more clearly distinguish the specific effects of DNS from the effects of general physical activity or attention. Second, combining biomechanical analysis with surface electromyography may help clarify the underlying neuromuscular mechanisms, particularly whether DNS training modifies muscle co-contraction patterns or activation strategies around the knee joint. Third, future studies should include validated clinical and functional outcome measures, such as WOMAC, KOOS, or stair-related functional scales, to determine whether biomechanical changes in COP control and lower extremity joint motion are accompanied by improvements in pain, physical function, or quality of life. Finally, future studies should consider using an extended staircase that allows continuous steady-state stair gait analysis and, where appropriate, comparing barefoot and shod conditions to better reflect real-life stair walking. Addressing these methodological issues may help further verify the biomechanical effects of DNS interventions in individuals with knee osteoarthritis.

## 5. Conclusions

The 10-week progressive DNS training program appeared to be feasible and safe for women with knee osteoarthritis, as indicated by the 100% retention rate, high adherence, and absence of adverse events. The intervention showed favorable preliminary changes in mediolateral COP control during stair ascent and descent, along with biomechanical changes characterized by increased sagittal-plane range of motion and reduced coronal-plane motion of the lower-limb joints. These findings suggest that DNS training may contribute to improved movement control strategies and dynamic stability during stair negotiation in women with knee osteoarthritis. However, given the pilot nature of the study and the small sample size, these results should be interpreted as preliminary. Future adequately powered randomized controlled trials are needed to confirm the effects of DNS training and to clarify its underlying biomechanical and neuromuscular mechanisms.

## Figures and Tables

**Table 1:** Baseline demographic and clinical characteristics of the participants (*n* = 26).

	DNSG (*n* = 13)	CG (*n* = 13)
Age (years)	56.77 ± 3.03	56.46 ± 3.64
Weight (kg)	60.18 ± 3.33	62.18 ± 6.32
Height (cm)	158.38 ± 4.91	158.25 ± 5.86
BMI (kg/m^2^)	24.01 ± 1.22	24.83 ± 0.61
Kellgren–Lawrence grade II, *n* (%)	8 (61.5)	7 (53.8)
Kellgren–Lawrence grade III, *n* (%)	5 (38.5)	6 (46.2)
VAS pain score of the index knee, 0–10	5.15 ± 0.80	5.46 ± 0.97
VAS pain score of less symptomatic knee, 0–10	3.46 ± 0.52	3.54 ± 0.97
Analgesic or anti-inflammatory medication use for knee pain at baseline, *n* (%)	3 (23.1)	4 (30.8)
Regular structured exercise participation at baseline, *n* (%)	0 (0.0)	0 (0.0)
Previous supervised rehabilitation experience or physical therapy for knee osteoarthritis, *n* (%)	10 (76.9)	9 (69.2)

Values are presented as means ± standard deviations. DNSG, dynamic neuromuscular stabilization group; CG, control group. Medication use refers to the self-reported intermittent or as-needed use of analgesic or anti-inflammatory medication for knee pain at baseline. Regular structured exercise participation refers to self-reported participation in planned exercise programs at baseline. Previous rehabilitation experience was defined as self-reported participation in supervised rehabilitation or physical therapy for knee osteoarthritis before study enrollment.

**Table 2:** The 10-week DNS-based exercise program.

DNS Training	Program	Time/Reps
Warm-up	1. Diaphragmatic breathing with functional IAP regulation	10/6
	2. Reciprocal cross-pattern activation in the 3-month supine position	
Main phase 1 (1–3 weeks)	1. Trunk rotation with core stabilization in lateral recumbent	35/8–10
	2. Anterior–posterior weight shifting in four-point kneeling	
	3. Unilateral hip extension with core stabilization in four-point kneeling	
Main phase 2 (4–7 weeks)	4. Asymmetrical kneeling transition with contralateral reach	35/8–10
	5. Contralateral reciprocal crawling pattern	
	6. Closed-kinetic chain stabilization in levitated four-point kneeling	
	7. Transition from four-point kneeling to unilateral lateral support	
Main phase 3 (8–10 weeks)	8. Transition to high plantigrade quadruped position for posterior chain elongation	35/8–10
	9. Unilateral lower extremity lift in plantigrade quadruped position	
	10. Transition from asymmetrical sitting to unilateral weight-bearing lunge	
	11. Closed-kinetic chain squatting from plantigrade quadruped posture	
Cool-down	1. Deep spinal flexion in quadruped resting position	5/6
	2. Prone resting with integrated respiratory-postural stabilization	

**Table 3:** Primary center of pressure outcomes after intervention.

Variable	Group	Baseline (M ± SD)	Post (M ± SD)	LSM (95% CI)	MD (95% CI)	*F*	*p*	η^2^_p_
ASC ML-velocity	DNSG	0.18 ± 0.04	0.15 ± 0.03	0.14 (0.14–0.15)	−0.05 (−0.06, −0.04)	84.34	<0.001	0.79
(normalized)	CG	0.18 ± 0.04	0.19 ± 0.03	0.19 (0.19–0.20)				
ASC ML-RMS	DNSG	1.01 ± 0.54	0.91 ± 0.49	0.94 (0.77–1.12)	−0.24 (−0.49, 0.01)	3.91	0.060	0.15
(cm)	CG	1.09 ± 0.67	1.21 ± 0.63	1.18 (1.00–1.35)				
DSC ML-velocity	DNSG	0.20 ± 0.05	0.16 ± 0.04	0.16 (0.14–0.17)	−0.05 (−0.07, −0.04)	37.37	<0.001	0.62
(normalized)	CG	0.19 ± 0.05	0.21 ± 0.04	0.21 (0.20–0.22)				
DSC ML-RMS	DNSG	0.76 ± 0.27	0.68 ± 0.20	0.69 (0.55–0.82)	−0.20 (−0.386, −0.004)	4.46	0.046	0.16
(cm)	CG	0.81 ± 0.34	0.89 ± 0.28	0.88 (0.75–1.02)				

Note. Baseline and post-intervention values are presented as mean ± standard deviation (SD). Adjusted means (least squares means, LSM) and 95% confidence intervals (CI) were calculated after controlling for baseline values as a covariate. MD = mean difference between groups (DNSG–CG). *F* and *p*-values were derived from analysis of covariance (ANCOVA). Partial eta squared (η^2^_p_) represents the effect size (0.01 = small, 0.06 = medium, 0.14 = large). DNSG = dynamic neuromuscular stabilization group; CG = control group; ASC = ascent; DSC = descent.

**Table 4:** Secondary center of pressure outcomes after intervention.

Variable	Group	Baseline (M ± SD)	Post (M ± SD)	LSM (95% CI)	MD (95% CI)	*F*	*p* (*p*-FDR)	η^2^_p_
ASC AP-velocity	DNSG	0.44 ± 0.08	0.45 ± 0.04	0.45 (0.43–0.46)	0.01 (−0.01, 0.03)	1.39	0.250	0.06
(normalized)	CG	0.45 ± 0.09	0.44 ± 0.06	0.44 (0.42–0.45)			(0.250)	
ASC AP-RMS	DNSG	3.63 ± 0.76	3.23 ± 0.84	3.26 (2.91–3.62)	−0.58 (−1.08, −0.07)	5.60	0.027	0.20
(cm)	CG	3.73 ± 0.68	3.88 ± 0.72	3.84 (3.49–4.20)			(0.054)	
DSC AP-velocity	DNSG	0.51 ± 0.08	0.49 ± 0.07	0.49 (0.45–0.53)	−0.04 (−0.09, 0.01)	2.30	0.143	0.09
(normalized)	CG	0.49 ± 0.08	0.52 ± 0.07	0.53 (0.49–0.56)			(0.143)	
DSC AP-RMS	DNSG	2.61 ± 0.87	2.47 ± 0.98	2.37 (1.96–2.78)	−0.59 (−1.18, −0.01)	4.43	0.047	0.16
(cm)	CG	2.24 ± 1.00	2.86 ± 0.72	2.96 (2.55–3.37)			(0.094)	

Note. Baseline and post-intervention values are presented as mean ± standard deviation (SD). Adjusted means (least squares means, LSM) and 95% confidence intervals (CI) were calculated after controlling for baseline values as a covariate. MD = mean difference between groups (DNSG–CG). *F* and *p*-values were derived from analysis of covariance (ANCOVA). Parenthesized values below *p*-values indicate FDR-adjusted *p*-values. Partial eta squared (η^2^_p_) represents the effect size (0.01 = small, 0.06 = medium, 0.14 = large). DNSG = dynamic neuromuscular stabilization group; CG = control group; ASC = ascent; DSC = descent.

**Table 5:** Secondary range of motion outcomes during stair ascent after intervention.

Variable	Group	Baseline (M ± SD)	Post (M ± SD)	LSM (95% CI)	MD (95% CI)	*F*	*p* (*p*-FDR)	η^2^_p_
Hip ROM (deg)	DNSG	61.59 ± 4.80	64.85 ± 5.66	65.32 (63.01–67.64)	4.01 (0.71, 7.30)	6.33	0.019	0.23
Saggital plane	CG	62.88 ± 4.13	61.80 ± 4.53	61.32 (59.00–63.64)			(0.029)	
Hip ROM (deg)	DNSG	18.58 ± 4.55	17.85 ± 3.95	17.25 (16.32–18.17)	−1.27 (−2.59, 0.05)	3.96	0.059	0.15
Coronal plane	CG	17.11 ± 5.56	17.91 ± 4.92	18.51 (17.59–19.44)			(0.071)	
Knee ROM (deg)	DNSG	52.45 ± 6.73	60.43 ± 9.55	60.86 (58.00–63.71)	9.70 (5.65, 13.74)	24.60	<0.001	0.52
Saggital plane	CG	53.30 ± 8.81	51.59 ± 9.01	51.16 (48.30–54.02)			(<0.001)	
Knee ROM (deg)	DNSG	31.83 ± 5.24	29.41 ± 4.07	29.93 (28.45–31.40)	−3.85 (−5.94, −1.75)	14.47	<0.001	0.39
Coronal plane	CG	33.07 ± 5.87	34.28 ± 6.14	33.77 (32.30–35.24)			(0.003)	
Ankle ROM (deg)	DNSG	15.81 ± 4.79	18.14 ± 5.68	18.96 (17.71–22.22)	2.92 (1.12, 4.72)	11.26	0.003	0.33
Saggital plane	CG	17.52 ± 4.08	16.87 ± 3.68	16.05 (14.79–17.31)			(0.006)	
Ankle ROM (deg)	DNSG	8.75 ± 2.12	7.50 ± 2.21	7.34 (6.35–8.33)	−0.37 (−1.76, 1.04)	0.29	0.595	0.01
Coronal plane	CG	8.25 ± 2.95	7.54 ± 2.51	7.71 (6.72–8.69)			(0.595)	

Note. Baseline and post-intervention values are presented as mean ± standard deviation. (SD). Adjusted means (least squares means, LSM) and 95% confidence intervals (CI) were calculated after controlling for baseline values as a covariate. MD = mean difference between groups (DNSG–CG). F and *p*-values were derived from analysis of covariance (ANCOVA). Parenthesized values below *p*-values indicate FDR-adjusted *p*-values. Partial eta squared (η^2^_p_) represents the effect size (0.01 = small, 0.06 = medium, 0.14 = large). DNSG = dynamic neuromuscular stabilization group; CG = control group; deg = degrees.

**Table 6:** Secondary range of motion outcomes during stair descent after intervention.

Variable	Group	Baseline (M ± SD)	Post (M ± SD)	LSM (95% CI)	MD (95% CI)	*F*	*p* (*p*-FDR)	η^2^_p_
Hip ROM (deg)	DNSG	15.40 ± 2.73	17.82 ± 3.64	18.48 (17.04–19.92)	2.81 (0.74, 4.89)	7.84	0.010	0.25
Saggital plane	CG	17.12 ± 3.57	16.32 ± 3.21	15.67 (14.22–17.11)			(0.020)	
Hip ROM (deg)	DNSG	18.91 ± 6.33	17.28 ± 5.31	16.42 (15.04–17.79)	−2.31 (−4.28, −0.35)	5.91	0.023	0.21
Coronal plane	CG	16.78 ± 3.83	17.87 ± 4.31	18.73 (17.35–20.10)			(0.028)	
Knee ROM (deg)	DNSG	47.95 ± 9.31	56.49 ± 7.40	57.74 (55.26–60.22)	9.87 (6.32, 13.41)	33.12	<0.001	0.59
Saggital plane	CG	51.41 ± 8.68	49.13 ± 8.07	47.87 (45.39–50.36)			(<0.001)	
Knee ROM (deg)	DNSG	28.47 ± 4.91	27.42 ± 3.63	27.86 (27.05–28.68)	−2.27 (−3.43, −1.12)	16.56	<0.001	0.42
Coronal plane	CG	29.52 ± 6.77	30.57 ± 6.33	30.14 (29.32–30.95)			(<0.001)	
Ankle ROM (deg)	DNSG	65.63 ± 5.75	68.82 ± 4.89	69.14 (66.86–71.41)	3.95 (0.73, 7.18)	6.43	0.018	0.22
Saggital plane	CG	66.63 ± 7.41	65.51 ± 6.46	65.19 (62.91–67.46)			(0.027)	
Ankle ROM (deg)	DNSG	6.55 ± 2.09	6.17 ± 2.02	6.22 (5.74–6.70)	−0.69 (−1.37, −0.02)	4.50	0.045	0.16
Coronal plane	CG	6.67 ± 3.51	6.96 ± 2.85	6.91 (6.44–7.39)			(0.045)	

Note. Baseline and post-intervention values are presented as mean ± standard deviation (SD). Adjusted means (least squares means, LSM) and 95% confidence intervals (CI) were calculated after controlling for baseline values as a covariate. MD = mean difference between groups (DNSG–CG). F and *p*-values were derived from analysis of covariance (ANCOVA). Parenthesized values below *p*-values indicate FDR-adjusted *p*-values. Partial eta squared (η^2^_p_) represents the effect size (0.01 = small, 0.06 = medium, 0.14 = large). DNSG = dynamic neuromuscular stabilization group; CG = control group; deg = degrees.

## Data Availability

The data presented in this study are available on request from the corresponding author.

## Supplementary Materials

The following supporting information can be downloaded at https://www.mdpi.com/article/10.3390/jfmk11030255/s1: Table S1: Supplementary Sagittal and Coronal Joint Moments during stair ascent; Table S2: Supplementary Sagittal and Coronal Joint Moments during stair descent.
