# Supplementary material for: Feasibility and Biomechanical Effects of Dynamic Neuromuscular Stabilization Training During Stair Negotiation in Middle-Aged Women with Knee Osteoarthritis: A Randomized Controlled Pilot Study
Source: J Funct Morphol Kinesiol. 2026 Jun 27;11(3):255. doi: 10.3390/jfmk11030255 (PMC13397932; doi:10.3390/jfmk11030255)
Supplement: Supplementary file 1 [file jfmk-11-00255-s001.zip › jfmk-4326255-supplementary.pdf]

Table S1. Supplementary Sagittal and Coronal Joint Moments during stair ascent

Variable	Group	Baseline (M±SD)	Post (M±SD)	LSM (95% CI)	MD (95% CI)	*F*	*P*	η^2_p
Hip moment (Nm/kg)	DNSG	0.88±0.20	0.93±0.16	0.93 (0.86-1.00)	-0.04 (-0.14, 0.07)	0.53	0.474	0.02
Sagittal plane	CG	0.88±0.17	0.96±0.19	0.96 (0.89-1.04)				
Hip moment * (Nm/kg)	DNSG	0.74±0.11	0.71±0.11	-	-0.09 (-0.19, 0.01)	-1.89	0.071	0.74
Coronal plane	CG	0.69±0.09	0.76±0.20	-				
Knee moment * (Nm/kg)	DNSG	0.58±0.17	0.54±0.10	-	-0.08 (-0.15, -0.00)	-2.20	0.037	0.86
Sagittal plane	CG	0.65±0.19	0.68±0.18	-				
Knee moment (Nm/kg)	DNSG	0.62±0.15	0.62±0.14	0.63 (0.58-0.68)	-0.02 (-0.10, 0.05)	0.43	0.520	0.02
Coronal plane	CG	0.64±0.13	0.66±0.12	0.65 (0.60-0.71)				
Ankle moment (Nm/kg)	DNSG	1.43±0.16	1.45±0.22	1.44 (1.36-1.53)	-0.05 (-0.18, 0.07)	0.72	0.405	0.03
Sagittal plane	CG	1.41±0.25	1.49±0.21	1.50 (1.41-1.58)				
Ankle moment * (Nm/kg)	DNSG	0.25±0.11	0.25±0.06	-	-0.01 (-0.08, 0.05)	-0.47	0.646	0.18
Coronal plane	CG	0.24±0.09	0.26±0.10	-				

Note. Baseline and post-intervention values are presented as mean ± standard deviation (SD). Adjusted means, expressed as least squares means (LSM), and 95% confidence intervals (CIs) were calculated after controlling for baseline values as a covariate. MD indicates the mean difference between groups (DNSG − CG). F and p values were derived from analysis of covariance (ANCOVA). Partial eta squared (η^2p) represents the effect size, with values of 0.01, 0.06, and 0.14 interpreted as small, medium, and large effects, respectively. DNSG = dynamic neuromuscular stabilization group; CG = control group; deg = degrees.
* Due to the violation of the homogeneity of regression slopes assumption, an independent t-test on the change scores (post minus baseline) was performed. For this variable, the values in the *F* and η^2p columns represent the *t*-value and Cohen's *d*, respectively, and the MD represents the mean difference between the change scores of the two groups

Table S2. Supplementary Sagittal and Coronal Joint Moments during stair descent

Variable	Group	Baseline (M±SD)	Post (M±SD)	LSM (95% CI)	MD (95% CI)	*F*	*P*	η^2_P
Hip moment (Nm/kg)	DNSG	0.59±0.23	0.50±0.21	0.50 (0.41-0.60)	-0.14 (-0.27, -0.01)	4.92	0.037	0.18
Sagittal plane	CG	0.61±0.10	0.65±0.13	0.64 (0.55-0.73)				
Hip moment (Nm/kg)	DNSG	0.94±0.17	0.92±0.14	0.92 (0.86-0.97)	-0.05 (-0.13, 0.03)	1.44	0.242	0.06
Coronal plane	CG	0.93±0.18	0.96±0.17	0.97 (0.91-1.02)				
Knee moment (Nm/kg)	DNSG	0.75±0.23	0.69±0.18	0.68 (0.63-0.73)	-0.09 (-0.16, -0.02)	7.53	0.012	0.25
Sagittal plane	CG	0.72±0.16	0.76±0.13	0.77 (0.72-0.82)				
Knee moment (Nm/kg)	DNSG	0.57±0.17	0.54±0.12	0.54 (0.51-0.58)	-0.05 (-0.10, 0.00)	4.01	0.057	0.15
Coronal plane	CG	0.59±0.09	0.60±0.08	0.59 (0.55-0.62)				
Ankle moment (Nm/kg)	DNSG	1.17±0.15	1.10±0.12	1.10 (1.05-1.15)	-0.11 (-0.18, -0.04)	9.38	0.006	0.29
Sagittal plane	CG	1.17±0.19	1.21±0.17	1.21 (1.16-1.26)				
Ankle moment (Nm/kg)	DNSG	0.18±0.09	0.19±0.08	0.19 (0.16-0.22)	-0.00 (-0.04, 0.04)	0.03	0.873	0.00
Coronal plane	CG	0.17±0.03	0.19±0.03	0.20 (0.17-0.22)				

Note. Baseline and post-intervention values are presented as mean ± standard deviation (SD). Adjusted means, expressed as least squares means (LSM), and 95% confidence intervals (CIs) were calculated after controlling for baseline values as a covariate. MD indicates the mean difference between groups (DNSG − CG). F and p values were derived from analysis of covariance (ANCOVA). Partial eta squared (η^2p) represents the effect size, with values of 0.01, 0.06, and 0.14 interpreted as small, medium, and large effects, respectively. DNSG = dynamic neuromuscular stabilization group; CG = control group; deg = degrees.
